# Thermal Properties’ Enhancement of PLA-Starch-Based Polymer Composite Using Sucrose

**DOI:** 10.3390/polym16081028

**Published:** 2024-04-09

**Authors:** Sri Yustikasari Massijaya, Muhammad Adly Rahandi Lubis, Rossy Choerun Nissa, Yeyen Nurhamiyah, Wida Banar Kusumaningrum, Resti Marlina, Riska Surya Ningrum, Jajang Sutiawan, Iman Hidayat, Sukma Surya Kusumah, Lina Karlinasari, Rudi Hartono

**Affiliations:** 1 Forest Products Department, Faculty of Forestry and Environment, IPB University, Bogor 16680, Indonesia; titamassijaya@gmail.com (S.Y.M.); karlinasari@apps.ipb.ac.id (L.K.); 2 Research Center for Biomass and Bioproducts, National Research and Innovation Agency, Bogor 16911, Indonesia; muha142@brin.go.id (M.A.R.L.); ross002@brin.go.id (R.C.N.); yeye001@brin.go.id (Y.N.); wida002@brin.go.id (W.B.K.); rest006@brin.go.id (R.M.); risk004@brin.go.id (R.S.N.); jaja007@brin.go.id (J.S.); iman005@brin.go.id (I.H.); sukm002@brin.go.id (S.S.K.); 3 Forest Products Department, Faculty of Forestry, Universitas Sumatera Utara, Medan 20353, Indonesia

**Keywords:** polylactic acid, sucrose, thermoplastic starch, biocomposite, glycerol

## Abstract

Polylactic-acid–starch-based polymer composite (PLA/TPS) has good thermal stability for biocomposites. However, the physical and mechanical properties of PLA/TPS do not meet the standards. It needed additives to enhance its physical and mechanical properties. The aim was to improve the physical and mechanical properties of PLA/thermoplastic starch using sucrose. In addition, this study evaluated the enhancement of thermal properties of PLA/thermoplastic starch using sucrose. This study used sucrose as an additive to enhance the PLA/TPS composite. The addition of sucrose inhibits the degradation of biocomposites. This means that thermal stability increases. The thermal stability increased because the degree of crystallinity increased with the addition of sucrose, which was also proven in the XRD result. The addition of sucrose caused the morphology of the biocomposite to have pores. The FESEM results showed that biocomposites with the addition of sucrose had pores and gaps. These gaps result from low adhesion between polymers, causing a decrease in the mechanical and physical properties of the sample. Based on the FTIR spectra, biocomposite PLA/TPS blends with the addition of sucrose still have many hydroxyl groups that will lead to attracting other molecules or ions, such as oxygen or water. This phenomenon affects the physical and mechanical properties of materials. The physical and mechanical properties increased with sucrose addition. The best composite was prepared using 3% sucrose. This is because sucrose has a crystalline structure that affects the properties of biocomposites. However, the addition of 3% sucrose was not as effective as that of neat PLA.

## 1. Introduction

Plastics are known to cause huge environmental problems, such as difficulty in degradation and environmental toxicity. Many researchers have developed biopolymer materials to replace conventional plastics [1,2,3]. Because of its good physical and mechanical properties, polylactic acid (PLA) is one of the most widely used biopolymers [4,5]. PLA is affordable compared to other biopolymers, such as polyhydroxyalkanoates (PHAs), poly hydroxybutyrate-valerate (PHBV), and poly (butylene succinate) (PBS) [1]. However, compared with conventional plastics, PLA still has high cost, high brittleness, and low heat resistance [1,6]. A material’s brittleness and thermal properties are related to its degree of crystallinity. Enhancing the crystallinity of a material can improve its properties [7]. Blending PLA with thermoplastic starch (TPS) has been reported to reduce the cost of PLA [8]. The blends of PLA/TPS (13.1%) have a higher degree of crystallinity than neat PLA/starch (5.8%). Starch and glycerol act as a nucleating agent in PLA, which increases its crystallization [9].

Thermoplastic starch (TPS) is a homogeneous starch blended with plasticizers [6]. Starch is affordable, easily degraded by microorganisms, readily available, and easy to process [8,9,10]. Cassava is a source of starch that can be transformed into tapioca flour. The amylose content in the tapioca flour (12.28–27.38%) can affect the mechanical properties. Glycerol has been used as a plasticizer [10]. Glycerol is a small molecule that can easily diffuse into starch polymers to disrupt hydrogen-bond interactions [11]. Adding glycerol as a plasticizer is desirable for improving the mechanical properties, especially Young’s modulus [10]. However, even with the addition of a plasticizer, the mechanical properties, particularly the tensile strength of PLA/TPS blends, are considered poor owing to the low interfacial adhesion between PLA/TPS [11]. Pantani and Turng [7] stated that additives can affect the characteristics of materials. An additive is a small quantity of material such as sucrose added to the composite and affects the characterization of composites. Sucrose is a nontoxic and renewable resource commonly found in various plants. Sucrose is a renewable, low-molecular-weight carbohydrate feedstock that can be an additive [12]. Alonso-Gonzalez et al. [13] found that sucrose can act as a filler and make the polymer bonds stronger. Massijaya et al. [14] found that adding 5% sucrose increased the WVTR value because sucrose lowered the homogeneity of the biocomposite. However, adding 5% sucrose increased the mechanical value of PLA/TPS.

Furthermore, biodegradation is also important for materials. Biodegradation is a chemical transformation by the presence of microorganisms in the environment, which relies on factors such as temperature, light, oxygen, humidity, and the molecular and chemical structure of the material. Massijaya et al. [14] found that 5% sucrose can delay biodegradation of PLA/TPS. In the present study, we attempted to make biocomposites with a lower sucrose content to make them more homogenous and biodegradable and improve their performance. Therefore, the aim was to study and improve the physical and mechanical properties of PLA/thermoplastic starch using sucrose. In addition, this study evaluated the enhancement of thermal properties of PLA/thermoplastic starch using sucrose.

## 2. Materials and Methods

### 2.1. Materials

Polylactic acid (PLA) in granule form was supplied from Prusa Polymers (Prague, Czech Republic). The melting point based on the specification of the PLA is 150–180 °C. Tapioca starch was supplied by PT. Umas Jaya Agrotama (Lampung, Indonesia). The plasticizer used in this study was glycerol, and the additive used was sucrose. Extra purity grade sucrose was purchased from MERCK (Jakarta, Indonesia). Glycerol was purchased from P&G Chemicals (Kuantan, Malaysia).

### 2.2. Biocomposite Preparation

There are two stages of blending to reach homogenous biocomposites. First, the thermoplastic starch and sucrose are blended. Second, TPS and PLA are blended. 

#### 2.2.1. Starch–Glycerol–Sucrose Blend

The materials used were sucrose, glycerol, and starch. The composition of materials is shown in Table 1. The sample (50 g) was blended for 10 min at 135 °C and a rotor speed of 80 rpm. The samples were blended using a twin-screw Haake Rheomix (Vreden, Germany). After the blending, 10 g of the sample was hot-pressed at 135 °C with a pressure of 10 MPa for 5 min. After the hot-pressing process, the sample became thermoplastic starch–sucrose (TPS-S). The TPS-S was then divided into small pieces and prepared for the next step.

#### 2.2.2. PLA-TPS Blend

The ratio of PLA to TPS was 60/40. The materials were blended using a twin-screw Haake Rheomix. The samples were blended for 10 min at 160 °C with a rotor speed of 80 rpm. After blending, 10 g of the sample was hot-pressed for 5 min at 160 °C with a pressure of 10 MPa.

### 2.3. Thermal Properties’ Analysis

The thermal properties of the PLA-TPS-S biocomposite were investigated using differential scanning calorimetry (DSC) and thermogravimetric analysis (TGA). TGA was performed using a TGA 4000 (Perkin Elmer Inc., Waltham, MA, USA) from 25 °C to 500 °C, at a heating rate of 10 °C/min. DSC analysis was performed using a DSC 4000 equipped with an intercooler (Perkin Elmer Inc., USA) from −20 °C to 250 °C at a heating rate of 10 °C/min.

### 2.4. Water Vapor Transmission Rate (WVTR)

WVTR quantifies the quantity of water vapor capable of permeating through a given material per unit area and time [14]. The WVTR measurement was performed by observing the weight of the sample eight times, referring to the TAPPI T464 om-12 standard [15]. The WVTR sample size was 3 × 3 cm. The sample was placed in a POL-EKO Apatura-type KK500 TOP+ INOX/G climatic chamber (POL-EKO, Wodzisław Śląski, Poland). The relative humidity (RH) was 90% at 25 °C. WVTR and water vapor permeability (WVP) were calculated using the following formula:

WVTRgm−2day=slope(g/s)A(m2)

where slope is the slope of the linear portion of the plot of weight gain versus time and A is the sample permeation area.

WVPgm.s.Pa=WVTR×L∆P

where L is the mean film thickness (m), and ∆P is the partial water vapor pressure difference between the two sides of the film (Pa).

### 2.5. Mechanical Strength 

The mechanical strength of the PLA-TPS-S biocomposite was tested using a UCT-5 Universal Testing Machine (Orientec Co., Ltd., Fukaya, Japan). The tests were based on ASTM D882-12, 2012 [16]. The sample was shaped like a dog bone. Before testing, the samples were measured using a micrometer screw. 

### 2.6. FTIR Analysis

Fourier-transform infrared (FTIR) spectroscopy was performed using an FTIR 4000 spectrometer (Perkin Elmer Inc., USA) with transmission mode. The spectra used were in the 4000–300 cm^−1^ frequency range. 

### 2.7. Crystallinity

The crystallinity was measured using X-ray diffraction (XRD) (Shimadzu 7000, Kyoto, Japan). θ = 40–60° with Cu radiation, a scan speed of 3°/min, and a voltage of 30 kV. The crystallinity was examined by dividing the total area of the crystalline peaks by the total area of the crystalline and amorphous peaks. 

### 2.8. Morphology Analysis

The morphology of the samples was captured using a scanning electron microscope (SEM) with a Thermo Scientific—Quattro (Waltham, MA, USA). The sample was cut transversely to approximately 0.05 cm and then coated with a thin layer of gold using sputtering. The samples were analyzed at 100× and 500× magnifications with an acceleration voltage of 10.00 kV and a high-vacuum detector.

### 2.9. Biodegradability Analysis

The study evaluated the biodegradability using ASTM G21 standards [17], employing salt agar as the growth medium and *Aspergillus niger* as the decomposing agent, as mentioned in past research by Nissa et al. [10]. Biocomposite was cut into 1.5 × 1.5 cm dimensions, and each experimental variation was replicated thrice. The observation period for the test was extended to more than 30 days. Before the test, all equipment was sterilized in an autoclave for 15 min. The initial culture was prepared by mixing *A. niger* with 10 mL sterile aquades, and 1 mL of this mixture was added to 9 mL sterile aquades to create a 10^−1^ culture. Subsequently, 1 mL of the 10^−1^ culture was added to 9 mL of sterile aquades to generate a 10^−2^ culture. From this 10^−2^ culture, 100 µL was transferred to a sterile Petri dish. Each Petri dish was supplemented with 20 mL salt agar and allowed to solidify. The biocomposite samples were placed on the agar surface. An even spread of 100 µL of 10^−2^ culture was applied to the samples. The growth of *A. niger* was quantified using ImageJ 2015 image processing software, presented by percent (%). 

## 3. Results

### 3.1. Thermal Properties

**Figure 1:**
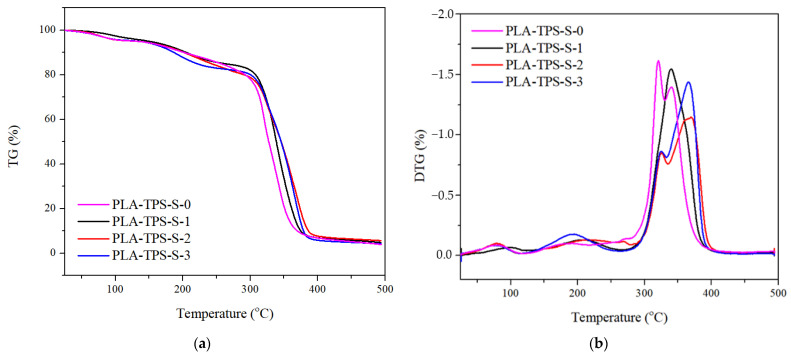
TGA curves (**a**) and DTG curves (**b**) of biocomposite with the sucrose addition of 0%, 1%, 2%, and 3%.

Figure 1 shows the TGA and derivative weight results for the sample. Figure 1a shows that the weight of the sample decreased as the temperature increased. This indicates that the sample was decomposed by heat. Table 2 presents T_onset_, T_max_, and weight loss (WL) of biocomposite. T_onset_ represents the temperature at which the weight loss begins, and T_max_ represents the temperature at which the weight loss ends. This study found that PLA-TPS-S-0 and PLA-TPS-S-1 have three peaks on the DTG curve, while PLA-TPS-S-2 and PLA-TPS-S-3 have four. All peaks represent the degradation of molecules.

The first peaks begin at a temperature of 54–82 °C and end at 77–97 °C (Table 2). At the first peak, the water molecule from glycerol evaporated [18], and 4.1–4.4% weight loss occurs. The second stages begin at 158–308 °C and end at 194–319 °C, when glycerol from TPS degrades [18]. Based on the study in [18], glycerol was expected to be degraded at 249 °C. Table 2 shows that glycerol degradation shifted to a lower temperature with the addition of sucrose. Kusumah et al. [19] reported that sucrose decomposition occurred at approximately 225 °C. Therefore, the addition of sucrose changes the temperature of decomposition of glycerol. The third peak begins at 311–333 °C, and the maximal temperature is 322–342 °C, at which starch degraded. Above 300 °C, the polymer chains, such as α-D-(1→4) from amylose and α-D-(1→6) from amylopectin glycosidic bonds [18], break, making the weight loss high (20–78%). PLA-TPS-S-2 and PLA-TPS-S-3 have a fourth peak that begins at 347–366 °C and ends at 367–371 °C. Figure 1 shows that the addition of sucrose inhibits the degradation of biocomposites. It was shown that at 309–311 °C, PLA-TPS-S-2 and PLA-TPS-S-3 degrade but then settle and have another degradation. This means thermal stability increases by adding more than 2% sucrose. The thermal stability increased because the degree of crystallinity increased with the addition of sucrose, which was also proven in the following XRD result. Pantani and Turng [7] explained that the additives could affect the flexibility, ductility, and processability of the polymer by decreasing degradation temperature of the polymer through reducing the intermolecular forces along polymer chains. 

**Figure 2:**
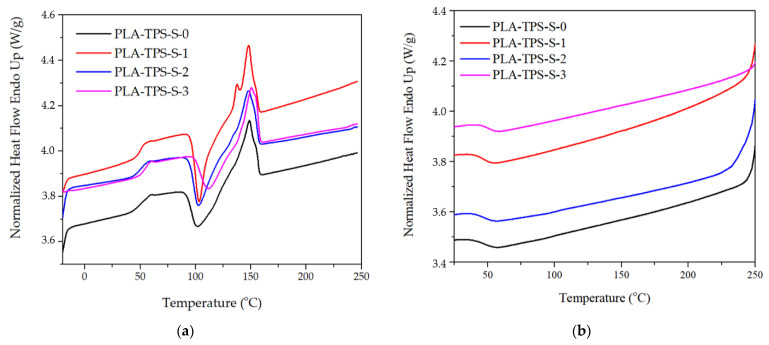
The DSC thermogram of heating (**a**) and cooling (**b**) of PLA-TPS-S-0,1,2,3.

DSC results are shown in Table 3 and Figure 2. Yang et al. [20] found the same double peak in biocomposite in the second heating. However, the present study observed it during the first heating. Several factors cause the double peak to occur: (1) the process of melting, recrystallization, and remelting when exposed to heat; (2) isodimorphism or polymorphism, which usually occurs in several crystal structures; (3) variations in lamellar thickness, arrangement, and shape; and (4) distinct molecular weights among different species [20]. Table 3 shows thermal parameters, including glass transition temperature (*T_g_*), melting temperature (*T_m_*), cold crystallization temperature (*T_cc_*), crystallization temperature (*T_c_*), melting enthalpy (∆*H_m_*), and crystallization enthalpy (∆*H_c_*).

The addition of sucrose can slightly increase the *T_g_*, *T_m_*, *T_cc_*_,_ as presented in Table 3. When the temperature rises, the polymer becomes amorphous and can mobilize, described as *T_g_*, and as the temperature increases, the molecular motion is enhanced [21]. The highest *T_g_* is shown by PLA-TPS-S-3 (55.22 °C), and the lowest is that of PLA-TPS-S-1 (49.62 °C). The following XRD also shows that PLA-TPS-S-1 has the smallest crystalline area, which leads to lower thermal properties [22]. Figure 2 shows that the peak of *T_m_* of PLA-TPS-S-1 widens, but as the sucrose content increases, the peak narrows. The study found that in the heating process of DSC, PLA undergoes self-nucleating before melting occurs, known as cold crystallization (*T_cc_*) [23]. *T_cc_* is the temperature transition from amorphous to crystalline form [21]. PLA-TPS-S-3 has the highest *T_cc_* (59.28 °C), as presented in Table 3. The highest ∆*H_m_* and ∆*H_c_* values are shown by PLA-TPS-S-2 (45.2 and −12). Kusumaningrum et al. [23] found that the ∆*H_m_* and ∆*H_c_* for neat PLA are 34.79 w/g and −15.33 w/g. It shows that adding sucrose affects the melting and crystallization of enthalpy (Table 3).

The crystallinity of a polymer is influenced by temperature, and when comparing its impact on material properties, it is crucial to conduct these studies at the same temperature. Typically, this is carried out at ambient temperature rather than at the melting point. As determined by DSC and XRD, the degree of crystallinity exhibited distinct results but displayed a similar trend. The values of crystallinity determined using XRD consistently exceeded those determined from DSC, as shown in Table 3 and Table 4. This occurrence occurred due to variations in the scanned regions of the identical sample. The XRD diffractograms solely examined the sample’s surface, whereas the DSC assessed the overall crystallinity of the sample. Hence, the injection molding process significantly impacted the crystallinity values seen in the XRD scan. The phenomenon caused changes in the structure and composition of the surface and shear zones, resulting in increased crystallinity due to the alignment of particles [24,25].

### 3.2. WVTR Characteristics

**Figure 3:**
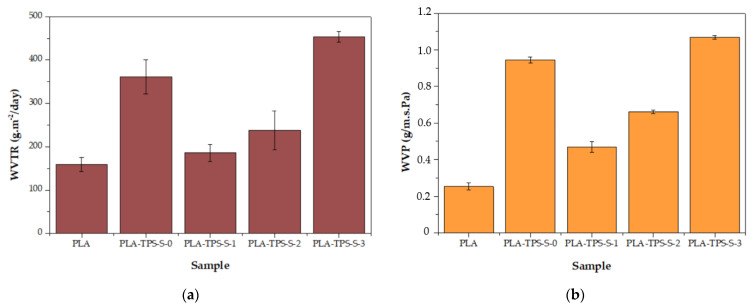
The WVTR (**a**) and WVP (**b**) value of PLA and PLA-TPS-S-0,1,2,3.

Figure 3 shows the results for the WVTR and WVP values. The WVTR measurement is used to quantify the quantity of water vapor that can pass through a material over a specific area and time [26]. Figure 3 shows that PLA-TPS-S-2 had a WVTR result approaching that of PLA, while PLA-TPS-S-3 had the highest WVTR result. It is known that materials with a porous structure morphology will facilitate water vapor transmission [26]. The morphology results show that the PLA-TPS-S-3 morphology is less homogeneous. The addition of sucrose caused the morphology of the biocomposite to have pores, which led to a decrease in the WVTR. Cazon et al. [27] explained that water can pass through a sample via diffusion. The permeability resulting from the interplay between solubility and diffusion is called the actual permeability. The transfer of water molecules through a sample involves a sequence of steps. The water molecule is initially transported towards the film interface and absorbed on the surface adjacent to the area with a higher water vapor concentration. Subsequently, the molecule dissolves, allowing diffusion to align with the driving force until complete dissolution occurs within the sample structure. Ultimately, the molecule is desorbed in the opposing side of the film and transported away from its surface [28,29,30]. In addition, the highest WVTR value is shown by PLA-TPS-S-3 due to sucrose’s water solubility. Sucrose is moderately soluble in water. At room temperature, approximately 276 g of sucrose can dissolve in 100 g of water [31]. Therefore, the presence of sucrose facilitates the transmission of water vapor.

The WVP measures the quantity of water penetrating the matrix per unit area, time, and pressure. Similar to the WVTR result, the WVP result also had the best performance for PLA, while PLA-TPS-S-2 approached the PLA’s WVP result. Studies have shown that adding hydrophilic low-molecular-weight plasticizers reduces polymer interaction, leading to higher molecular mobility and allowing water migration [32]. This makes the polymer interactions dense and a large free volume, leading to higher water diffusion through the sample [33]. In this case, PLA-TPS-S-3 has more plasticizer content, which leads to a higher WVP. 

### 3.3. Mechanical Characteristics

**Figure 4:**
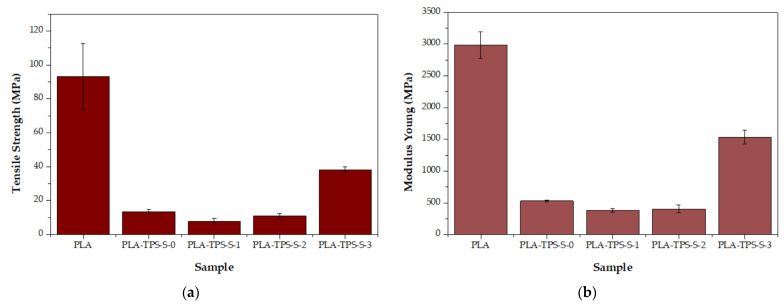
Tensile strength (**a**) and Young’s modulus (**b**) of PLA and PLA-TPS-S-0,1,2,3.

A comparison of the Young’s modulus and tensile strength values between each sample is presented in Figure 4. The Young’s modulus quantifies the strength of interatomic bonds and is influenced only by the slight microstructural configuration of the materials [34]. Neat PLA (2983 MPa) exhibited the best Young’s modulus value among the samples. Figure 4 shows that the average of PLA-TPS-S-2 (405.09 MPa) is slightly higher than that of PLA-TPS-S-1 (384.86 MPa). It was also shown that Young’s modulus increased with the addition of sucrose and in PLA-TPS-S-3, which approached neat PLA’s mechanical strength value. Based on this study, glycerol can reduce the interaction between chains in the material, which increases the mobility of the polymer chain and the viscoelastic response, leading to higher flexibility [32,35]. Another study by Agwamba [9] found that Young’s modulus can perform better as the number of hydroxyl groups increases. Based on the FTIR result, sucrose has many hydroxyl groups that affect the performance of the Young’s modulus. 

The tensile strength is the maximum strength of the film until it breaks [35]. The tensile strength of neat PLA is 93.14 MPa. In comparison, that of PLA-TPS-S-0 is 13.54 MPa and that of PLA-TPS-S-3 is 38.27 MPa (Figure 3). This large decrease in tensile strength is because starch, glycerol, and sucrose have a large number of hydroxyl groups that cause dispersion. The interaction between the polymer chains becomes weak [35,36,37]. As mentioned previously, the FTIR result of the biocomposites shows many hydroxyl groups. Another study by Fadini et al. [32], which blended sucrose and collagen, showed a different result as the addition of sucrose increased and the tensile strength decreased. Figure 4 shows that the tensile strength increased as the sucrose content increased. This indicates that other materials that blend with sucrose affect the effect of sucrose on tensile strength.

### 3.4. FTIR Spectra

**Figure 5:**
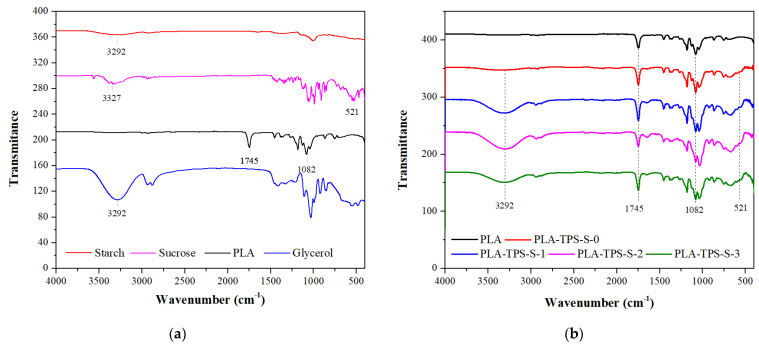
FTIR Spectra of starch, sucrose, PLA, and glycerol (**a**) and PLA-TPS-S-0,1,2,3 (**b**).

FTIR is an instrument used to identify molecular structures with spectra that can specifically describe chemical bonds to estimate the type of interaction that occurs after blending materials [38,39]. Figure 5 shows the FTIR spectra of the biocomposite samples with the addition of sucrose. Yang et al. [20] found that neat PLA has a stretching vibration of C=O bonds (1745 cm^−1^). Orskov et al. [40] found starch peaked at 450–707, 1082, 1380, and 3600, which indicates starch has a skeletal mode of pyranose ring, C-O-H bending, C-H and C-O-H deformation, and carbohydrate OH stretching. Starch has many hydroxyl groups, which can lead to poor mechanical properties because of the weak molecular force between the polymer chains [35,36,41]. Figure 5 shows that the spectra of biocomposites have a wide peak at 450–707 cm^−1^ and peaks at 1082 and 1380 cm^−1^. Figure 5b shows that sucrose formed a new stretch vibration, with a wider peak at 3000–3600 cm^−1^. Based on the FTIR spectra, biocomposite PLA/TPS blends with the addition of sucrose still have many hydroxyl groups that will lead to attracting other molecules or ions, such as oxygen or water [42]. This phenomenon affects the physical and mechanical properties of materials.

### 3.5. XRD 

**Figure 6:**
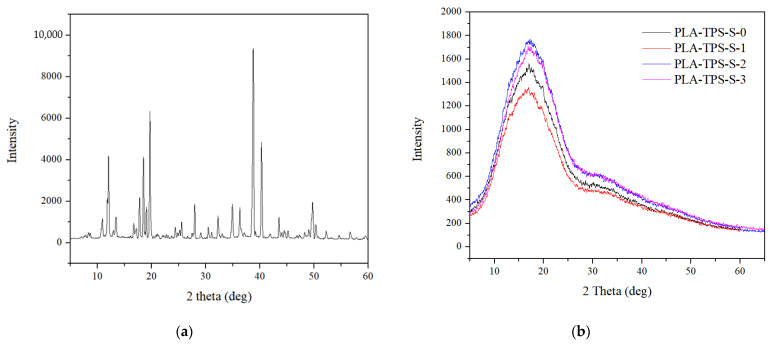
XRD pattern of sucrose (**a**) and PLA-TPS-S-0,1,2,3 (**b**).

XRD can determine the phase structure, purity, and crystallinity of a material’s phase structure, purity, and crystallinity [43]. The XRD patterns of sucrose are presented in Figure 6a, and the biocomposites are presented in Figure 6b. The XRD pattern (Figure 6a) shows a broad and wide peak representing the amorphous structures of biocomposites. No peak shifts are observed. All samples showed a peak at approximately 17.04°. PLA peak 2θ is 16.2° while thermoplastic starch 2θ is 20°. This indicates that the peak is shifting because of the blending. According to another study [44], PLA blended with PEG peaks at approximately 16.8°, representing an amorphous microstructure. Sucrose has a crystalline structure (Figure 6b). The presence of sucrose shows a good impact by making the crystalline area wider for PLA-TPS-S-2 and PLA-TPS-S-3 (Figure 6b). As indicated by the thermal properties (Figure 1), PLA-TPS-S-2 and PLA-TPS-S-3 exhibited higher decomposition temperature than PLA-TPS-S-0. However, Table 4, presenting the data on crystallinity degree, shows otherwise. PLA-TPS-S-0 has a bigger degree of crystallinity.

**Table 4:** Degree of crystallinity of PLA-TPS-S-0,1,2,3 obtained by XRD.

Sample	Degree of Crystallinity (%)
Sucrose	52.73
PLA	53.07
PLA-TPS-S-0	40.99
PLA-TPS-S-1	44.93
PLA-TPS-S-2	47.81
PLA-TPS-S-3	46.96

### 3.6. Morphology

**Figure 7:**
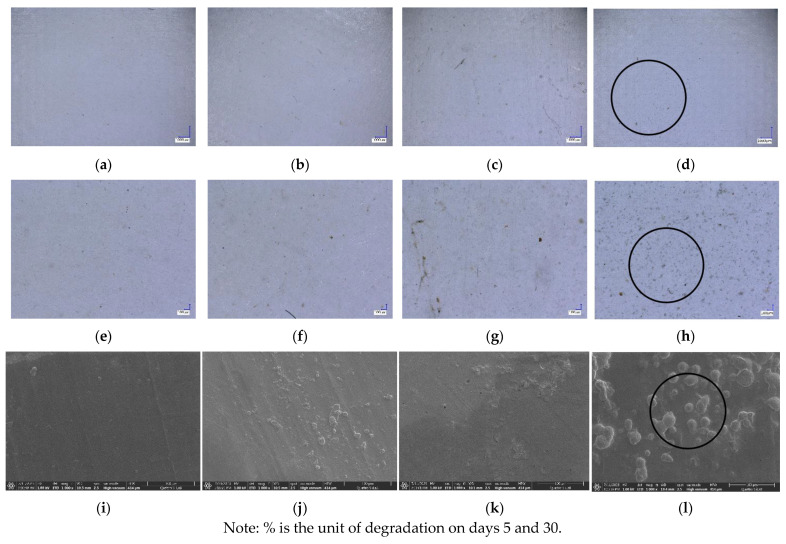
Morphology of PLA-TPS-S-0,1,2,3 taken by digital microscope with magnification 20× (**a**–**d**) and magnification 100× (**e**–**h**). Morphology of PLA-TPS-S-0,1,2,3 taken with FESEM (**i**–**l**).

The morphology of biocomposites was captured using digital microscope from Keyence (Osaka, Japan) and FESEM, as shown in Figure 7. Differences in morphology were observed for each sample. Figure 7b–d show sucrose particles that cannot be degraded by heat and become aggregates. Occasionally, aggregation occurs because of the interaction force between the filler and the matrix [45,46,47]. The FESEM results showed that biocomposites with the addition of sucrose had pores and gaps. These gaps result from adhesion between polymers, causing a decrease in the mechanical and physical properties of the sample [48].

### 3.7. Biodegradability

Biodegradation is a chemical transformation by the presence of microorganisms in the environment, which relies on factors such as temperature, light, oxygen, humidity, and the molecular and chemical structure of the material [10]. The growth of *A. niger* on the sample indicated that *A. niger* used the sample as the sole source of carbon [12], indicating the biodegradability of the sample. Ferrarezi et al. [8] stated that if PLA is exposed to the environment, it can break down into carbon dioxide, methane, and water over a few months to two years. Time, temperature, low-molecular-weight contaminants, and catalyst concentration all affect how quickly it degrades. It is easy for *A. niger* to be cultivated on organic materials such as litter media and decomposing plants. Massijaya et al. found biocomposites without the addition of sucrose makes *A.niger* grown easier (approximately 69.47%). This indicated that *A. niger* is easier to culture in the presence of sucrose. Another study using sucrose blended with LDPE reported different results. The addition of sucrose accelerated *A. niger* growth. This indicates that PLA degrades more easily than LDPE does.

The large colonies of *A. niger* indicate that the more easily a sample is consumed by *A. niger,* the more easily the sample is degraded. Table 5 shows the growth of *A. niger* in biocomposite samples. PLA-TPS-S-2 showed the highest growth of *A. niger* (38%) because the moisture content of PLA-TPS-S-2 was the lowest (8.7%) (Table 6). The other samples had a high moisture content. Particle aggregation, matrix porosity, air-filled porosity, and matrix gas permeability are all affected by high moisture content, which could affect the delivery of vital oxygen into the composting zone where carcass decomposition occurs [49].

## 4. Conclusions

The addition of sucrose inhibits the degradation of biocomposites. The thermal stability increased because the degree of crystallinity increased with the addition of sucrose, which was also proven in the XRD result. The addition of sucrose caused the morphology of the biocomposite to have pores. The FESEM results showed that biocomposites with the addition of sucrose had pores and gaps. These gaps result from low adhesion between polymers, causing a decrease in the mechanical and physical properties of the sample. Based on the FTIR spectra, biocomposite PLA/TPS blends with the addition of sucrose still have many hydroxyl groups that will lead to attracting other molecules or ions, such as oxygen or water. This phenomenon affects the physical and mechanical properties of materials. The physical and mechanical properties increased with sucrose addition. The best composite was prepared using 3% sucrose. This is because sucrose has a crystalline structure that affects the properties of biocomposites. However, the addition of 3% sucrose was not as effective as that of neat PLA.

## Figures and Tables

**Table 1:** Composition of the prepared biocomposites.

Stage	Sample	Starch (%)	Glycerol (%)	Sucrose (%)
First Blending	TPS-S-0	65	35	0
	TPS-S-1	64.5	34.5	1
	TPS-S-2	64	34	2
	TPS-S-3	63.5	33.5	3
**Stage**	**Sample**		**PLA (%)**	**TPS-S (%)**
Second Blending	PLA-TPS-S-0		60	40
	PLA-TPS-S-1		60	40
	PLA-TPS-S-2		60	40
	PLA-TPS-S-3		60	40

**Table 2:** Value of thermal stability parameters based on TGA and DTG of PLA-TPS-S-0,1,2,3.

Peak	Sample	T_onset_ (°C)	T_max_ (°C)	WL (%)
1	PLA-TPS-S-0	58.06	77.02	4.4
	PLA-TPS-S-1	82.24	97.76	4.1
	PLA-TPS-S-2	57.12	78.81	4.4
	PLA-TPS-S-3	54.68	77.06	4.2
2	PLA-TPS-S-0	308.65	319.62	39.8
	PLA-TPS-S-1	174.06	203.83	10.4
	PLA-TPS-S-2	157.04	210.47	12
	PLA-TPS-S-3	158.78	194.79	13
3	PLA-TPS-S-0	333.97	342.86	40.8
	PLA-TPS-S-1	317.34	340.14	78.1
	PLA-TPS-S-2	311.74	339.76	21.7
	PLA-TPS-S-3	309.08	322.30	20.4
4	PLA-TPS-S-0	-	-	-
	PLA-TPS-S-1	-	-	-
	PLA-TPS-S-2	366.42	371.76	49.6
	PLA-TPS-S-3	347.39	367.86	54.7

**Table 3:** The value of thermal parameters and crystallinity of PLA-TPS-S based on DSC analysis.

Sample	*T_g_* (°C)	*T_m_* (°C)	*T_cc_* (°C)	∆*H_m_* (J/g)	∆*H_c_* (J/g)	X_c_ (%)
PLA-TPS-S-0	51.91	148.63	58.46	38.5	−11.75	27.86
PLA-TPS-S-1	49.62	148.13	57.29	40.6	−11.70	30.10
PLA-TPS-S-2	50.93	147.63	59.30	45.2	−12.00	34.58
PLA-TPS-S-3	55.22	150.31	59.28	39.01	−11.14	29.03

**Table 5:** Day 5 and Day 30 of biodegradability by *A. niger* test.

Day	PLA-TPS-S-1	PLA-TPS-S-2	PLA-TPS-S-3
5	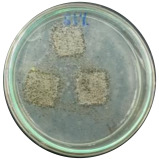	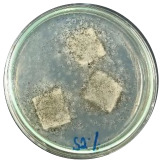	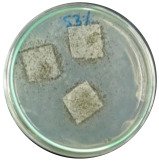
	15.5%	18%	16.2%
30	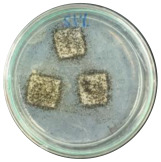	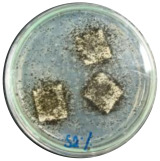	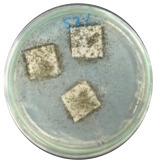
	59.9%	72%	65.2%

Note: % is the degradation unit at Day 5 and Day 30.

**Table 6:** Moisture content of PLA-TPS-S-1,2,3.

Sample	Moisture Content (%)	Standard Deviation
PLA-TPS-S-1	10.4	1.52
PLA-TPS-S-2	8.7	1.12
PLA-TPS-S-3	11.2	0.28

## Data Availability

The data presented in this study are included in the article or have been made available through the Supplementary Materials.

## Supplementary Materials

The following supporting information can be downloaded at https://www.mdpi.com/article/10.3390/polym16081028/s1, Excel file: Raw data.
